# Protocol for mapping psychosocial screening to resources in pediatric oncology: a pilot randomized controlled trial

**DOI:** 10.1186/s40814-021-00878-0

**Published:** 2021-07-17

**Authors:** Leandra Desjardins, Kelly Hancock, Peter Szatmari, Sarah Alexander, Wendy Shama, Claire De Souza, Denise Mills, Oussama Abla, Maru Barrera

**Affiliations:** 1 Charles-Bruneau Cancer Center, Sainte-Justine University Health Center, Montreal, Canada; 2 Department of Psychiatry, Hospital for Sick Children, Toronto, ON Canada; 3 Division of Haematology/Oncology, Hospital for Sick Children, Toronto, ON Canada; 4 Department of Social Work, Hospital for Sick Children, Toronto, ON Canada

**Keywords:** Screening, Pediatric oncology, Mental health, Psychosocial, Protocol

## Abstract

**Background:**

A pediatric cancer diagnosis and its treatment can have a detrimental effect on the mental health of children and their families. Screening to identify psychosocial risk in families has been recognized as a standard of care in pediatric oncology, but there has been limited clinical application of this standard thus far. A significant impediment to the implementation of psychosocial screening is the dearth of information on how to translate psychosocial screening to clinical practice, and specifically, how to follow-up from screening results. This manuscript aims to describe a protocol of a new intervention examining the feasibility and acceptability of mapping via a Psychosocial Navigator (PSN) psychosocial screening results to specific recommendations of resources for families based on measured risk for psychosocial distress and mental health symptoms.

**Methods:**

The pilot randomized control trial (RCT) consists of dyads of youth (10–17 years) newly diagnosed with cancer and their primary caregiver. This RCT includes two arms (intervention and control group), with each group completing measurements near diagnosis and 1 year later. After the initial assessment, dyads in the intervention group receive monthly screening results and recommendations from the study PSN that are tailored to these results. The patient’s primary healthcare team (nurse, social worker, oncologist) also receive the risk, distress, and mental health results as well as the recommendations from the PSN.

**Discussion:**

This study addresses a significant barrier to the implementation of psychosocial screening in pediatric oncology: specifically, the limited knowledge of how to follow-up from screening results. Findings from this pilot will inform a future definitive RCT to test the effectiveness of the intervention on patient and family mental health outcomes. This project has implications for enhancing clinical care in pediatric oncology, as well as other pediatric populations.

**Strengths and limitations of this study:**

This is the first study of screening and follow-up using a psychosocial navigator.

This study involves both patient and caregiver report.

The small sample size necessitates a future larger study to investigate the effects of intervention.

**Trial registration:**

NCT04132856, Registered 10 October 2019—retrospectively registered.

## Background

Approximately 300,000 children worldwide are diagnosed with cancer annually [1]. A pediatric cancer diagnosis and its treatment can have detrimental mental health effects on children/adolescents (youth) and their families, and subsequently on their quality of life [2, 3]. Youth diagnosed with cancer have more problems related to depression and anxiety than their healthy peers [4], and these symptoms are often present early in the diagnosis and treatment trajectory [5, 6]. Pre-teens and adolescents with cancer, in particular, have been identified as being at greater risk for mental health problems and reduced health-related quality of life [7, 8] and yet they have also been identified as an underserved group [9]. Caregivers of children diagnosed with cancer have been found to experience elevated symptoms of anxiety and depression, particularly near diagnosis and during the active treatment period [5, 10–12].

**Fig. 1:**
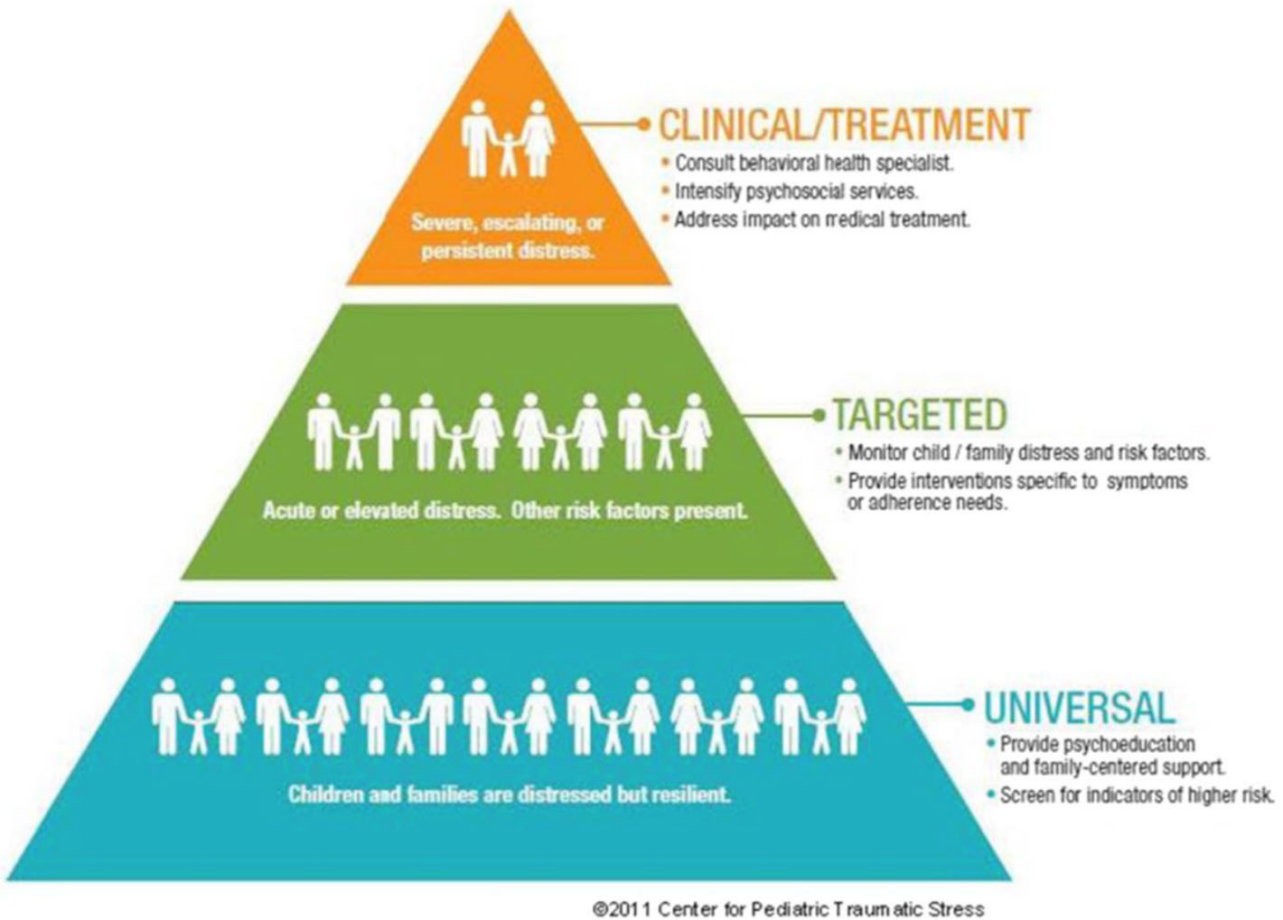
The Pediatric Preventive Psychosocial Health Model (PPPHM)

Of the 15 standards of psychosocial care in pediatric oncology [13], systematic psychosocial screening of families has received the greatest empirical support [14]. Early screening for psychosocial risk and mental health symptoms in youth with cancer and their family is an important first step for (1) identifying individuals and families who may be at risk for poor mental health and psychosocial adjustment; and (2) indicating a need for early psychological intervention and follow-up to prevent mental health impairments in this population [15]. The Psychosocial Assessment Tool (PAT) is an evidence-based instrument designed as a parent report measure for pediatric cancer and is grounded on the Pediatric Preventive Psychosocial Health Conceptual Model (PPPHM) of psychosocial risk (see Fig. 1) [16, 17]. Psychosocial risk is defined as “a constellation of individual, family, social, and economic factors that, when considered collectively, increase the likelihood that an individual or family members will experience difficulties in managing the challenges of cancer and its treatment” [18]. On the basis of the PPPHM, families are categorized into one of three risk levels: *universal*, representing the majority (~ 50–70%) of families who present minimal family risk factors and sufficient resources when facing childhood cancer; *targeted* families (~ 30%), who endorse some areas of risk and moderate resources, and, accordingly, may benefit from targeted (e.g., short-term, focused) interventions to assist with their adjustment; and finally, *clinical* families (~ 10–15%), who have more severe problems, many risk factors, few resources, and pre-existing mental health issues, and consequently likely require more intensive interventions to support their adjustment to pediatric cancer (see Fig. 1) [16, 17].

Unfortunately, a limited number of pediatric institutions have implemented standardized psychosocial screening [19, 20] and even less evidence exists of the impact of psychosocial screening on patient and family outcomes [21]. The greatest impediment to the effective use of screening tools to assess psychosocial risk and distress in pediatric oncology remains the dearth of information on how to translate screening to clinical practice [22, 23]. Using the PAT, validated and adapted for the Canadian population [24], our team previously conducted a pilot *randomized controlled trial* (RCT) examining (a) the feasibility of conducting psychosocial screening in pediatric oncology using the PAT and providing a summary of the PAT to the child’s primary medical team, and (b) preliminary patient outcomes. Acceptability of PAT among providers was variable, with nurses reporting greater utility followed by oncologists and social workers reporting the lowest utility [25]. Child’s proxy pain-related quality of life (QOL) *(n*^*2*^ *= 0.08)* appeared to improve to a greater extent in the intervention compared to the control group [21]. After feedback from health care providers regarding the PAT summary and examining barriers to psychosocial screening [22], a larger (caregiver n = 122), definitive randomized controlled trial was conducted examining the effects of providing a psychosocial risk summary (revised) to the primary care team (oncologist, nurse, social worker) of a child newly diagnosed with cancer. Compared to the control group, improvements were seen over time in the intervention group on the patient proxy-reported QOL measure (*n*^*2*^ *= 0.87)* within the targeted/clinical risk subgroup [26]. As well, less depression symptoms were found in caregivers in the intervention group (d = .60) compared to the control group within the targeted/clinical risk [27]. Regardless of the group allocation, depression (d = .59) and anxiety symptoms (d = .47) declined over time for the caregivers; self-reported anxiety symptoms declined over time for siblings (d = .99), but neither depression nor anxiety self-reported symptoms declined over time in patients. Aside from the fact that the patient and sibling self-report samples were underpowered, these findings generally suggest that sharing early psychosocial risk screening results with the treating team is helpful but not enough to have a larger effect on mental health and quality of life outcomes in the family.

These previous studies [21, 24, 26, 27] were the foundation for the current pilot randomized control trial of an enhanced intervention. Specifically, the screening intervention was enhanced by including a psychosocial navigator with a unique role of communicating screening risk and mental health results initially and monthly for a year, tailoring resources recommendations to screening results, and communicating the screening information to both families and healthcare providers. We suspected these improvements may strengthen intervention impact on mental health and quality of life outcomes *in both* youth and the caregiver separately.

Further variables of interest will also be explored in this study, in order to determine outcomes to be further examined in a larger definitive RCT. Although coping strategies have not been examined in the context of psychosocial screening intervention, how people cope have been found to be critical for their psychosocial adjustment: primary control coping (e.g., problem solving, emotional modulation) and secondary control coping (e.g., acceptance, cognitive reappraisal) are associated with better psychosocial adjustment in caregivers of children diagnosed with cancer [28]. Thus, coping strategies are explored in this pilot study as potential mediators. Another important domain in the child’s life that is typically impacted by cancer diagnosis and treatment is social relations with friends and peers [29]. Thus, changes in social relations related to the intervention are also examined as exploratory outcomes. Finally, considering the well documented impact of childhood cancer treatment on the child fatigue level, sleep quality, and pain [30–32], these factors will also be explored as potential outcomes of this intervention. To summarize, this pilot RCT will inform if it is possible to conduct this enhanced screening intervention and which outcome variables appear to be related to the intervention. This information will be used for planning the future, definitive RCT evaluating the enhanced intervention with a large multi-center RCT.

## Aims and objectives

The purpose of this manuscript is three-fold: (a) to describe a comprehensive screening/intervention program called Enhanced Psychosocial Screening Intervention (EPSI), which was developed based on our previous work and feedback from health care providers and caregivers; (b) to conduct a pilot RCT to determine the feasibility and acceptability of EPSI to reduce caregiver and patient mental health symptoms and improve their quality of life. Primary outcomes are feasibility and acceptability of recruitment and data collection methods, acceptability of intervention components and delivery. Secondary outcomes are anxiety and depression symptoms and quality of life. Exploratory outcomes are measures of coping, social relationships, and physical outcomes (fatigue, sleep quality, and pain).

## Methods/design

### Study design

**Fig. 2:**
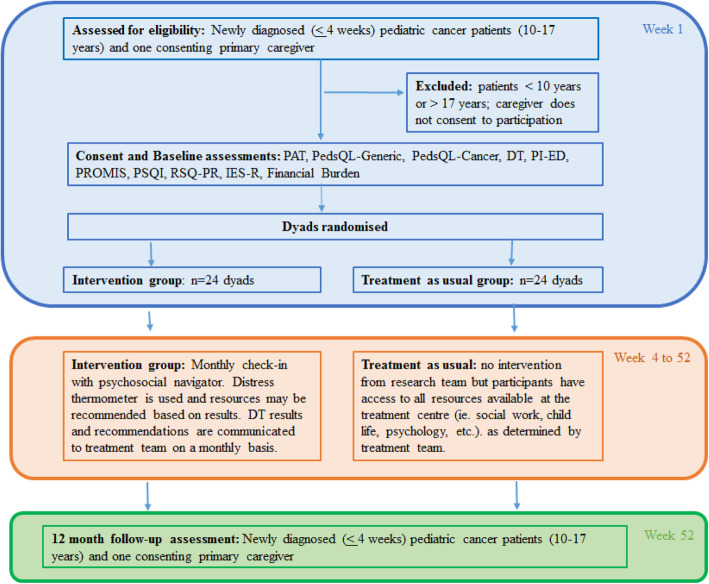
CONSORT flow chart

We use a parallel, randomized controlled pilot trial design with two study arms: the enhanced psychosocial intervention group (IG) and the treatment as usual control group (CG). Outcome assessments are conducted at baseline (2–6 weeks from diagnosis) and 12 months later (see Fig. 2 for timeline). Prior to the initiation of this study, we consulted with key stakeholders (community parents of children with cancer organization, oncologists, nurses, social workers, psychiatrists) regarding study design. We plan to continue to share outcomes of the study with stakeholders in order to foster continued engagement in psychosocial screening as a process.

### Randomization

Upon completion of baseline measures, participants are randomly allocated at a 1:1 ratio to either the IG or CG. Using computer-generated numbers, the randomization is managed by the research coordinator for the project. The study coordinator maintains a double password-protected file with details of randomized allocation and reveals the randomization outcome to PSN after dyads complete consent procedures.

### Blindness

A research assistant who is blinded to participant group allocation and study objectives scores outcome measures. Although healthcare providers (HCPs) caring for the child in the IG are not blinded to group allocation, HCPs in general are blinded to the identity of the participants in the CG.

### Study setting

Every year, approximately 600 youth are diagnosed with cancer in Ontario. Of these, approximately 60% of all pediatric oncology patients are treated at the Hospital for Sick Children (HSC), with an estimate of approximately 100 youth between the ages of 10 and 17 years. We aim to recruit approximately 40 dyads (one caregiver and youth) over a 16-month period.

As the largest pediatric academic health sciences center in Canada at the forefront of pediatric research and treatment, HSC is an ideal location to conduct this research project. Given the low incidence rate of pediatric cancer, the extensive catchment area served by HSC will be instrumental for recruitment of the necessary sample size. HSC provides a supportive, extensive research infrastructure including biostatistics, design, and analysis services. Additionally, within the oncology program, we have a collaborative well-established team of pediatric oncology clinician/researchers with rich psychosocial expertise.

### Participant inclusion criteria

The study is conducted with youth with cancer and one caregiver (primary caregiver). *Inclusion criteria for youth with cancer*: a new diagnosis of any type of cancer (within 4 weeks of diagnosis), between the ages of 10 and 17 years, medically suitable for participation (determined by the patient’s oncologist) and in active treatment (i.e., not palliative). Youth were excluded only if the treating clinical team thought a psychiatric or developmental disorder prevented them from active participation (e.g., completing assessment measures). *Inclusion criteria for caregiver*: primary caregiver of youth with cancer (as outlined above). Both youth with cancer and caregiver must agree to participate in the study. Given the culturally diverse population at HSC, families with limited English fluency but interested in participating are provided with translation services.

### Participant exclusion criteria

*Exclusion criteria for youth with cancer*: a diagnosed psychiatric, physical, or developmental disorder which prevents him/her from completing assessment measures; is considered not medically suitable for participation (as determined by the treating oncologist); primary caregiver does not participate. *Exclusion criteria for caregiver*: does not self-identify as the primary caregiver; youth with cancer does not participate.

### Recruitment procedure

Potential participants are identified by the treatment team and central database. Those who meet the eligibility criteria receive a brief synopsis of the study by a health care professional that is known to them. If they are then interested in hearing more about the study, a trained research assistant meets with them to review the study in more detail, verbally guides them through the written consent form, and provides some time for the participant to review it independently. If patient and caregiver decide to participate they sign the written consent form in the presence of the research assistant. Patient and caregiver are consented separately. Participation is voluntary and dyads are informed that they may withdraw at any time. After the consent form is signed, the baseline measures are completed and the family is randomized. A similar assessment battery is completed 12 months later. Each participant (patient and caregiver in each family) receives a $25 gift card at baseline and completion of study as an expression of gratitude for their participation.

### Sample size

As recommended for pilot RCT [33], enrolling 36–40 consenting patients-caregiver dyads, 18–20 in each group, will be acceptable to achieve the study objectives (see Fig. 2, participant flow chart).

### Intervention

#### CG

This group receives available psychosocial care services at the institution; these services (e.g., child life, psychology, art and music therapy, and psychiatry) are accessible to both CG and IG groups based on the clinical judgement of the core team. The core team consists of an oncologist, a social worker, and a nurse.

#### IG

**Fig. 3:**
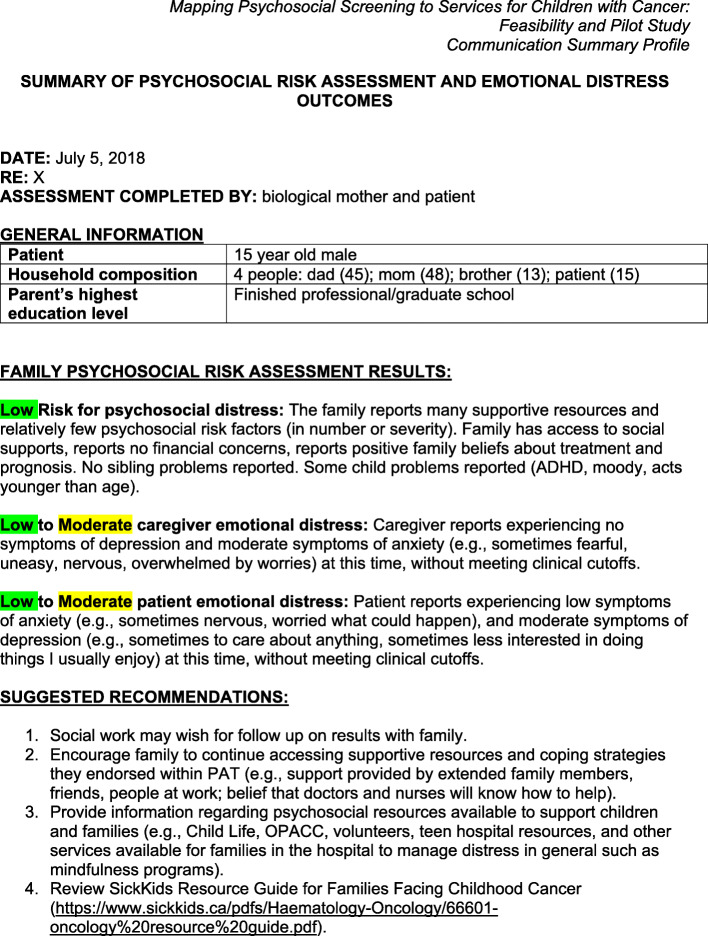
Example of Baseline Communication Summary Profile for a Fictional Patient

**Fig. 4:**
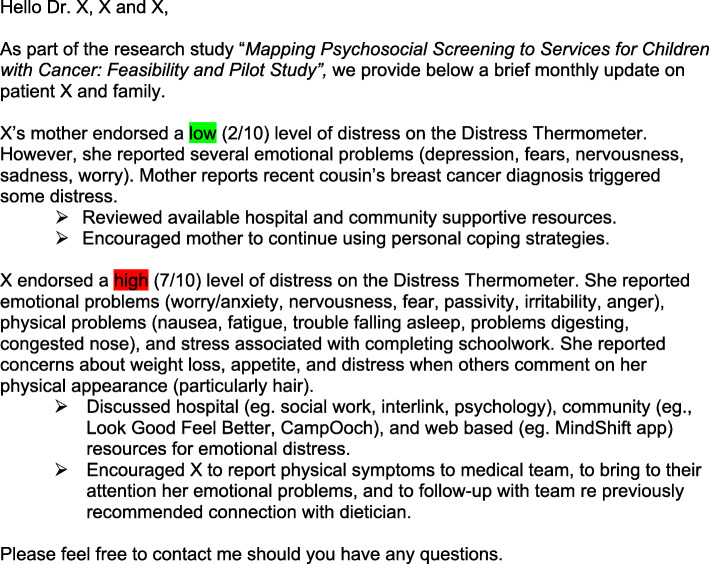
Example of Monthly Distress Thermometer Communication for a Fictional Patient

The IG receives whatever available services the treating team recommends, as described above, and the EPSI services on a monthly basis for 1 year. EPSI consists of the following components: (1) translating screening for *psychosocial risk, distress, and mental health* assessments results at baseline into intervention recommendations, mapped to resources based on levels of psychosocial risk (PAT: universal, targeted, clinical) and levels of depression and anxiety (mild, moderate, and high), as determined by the standardized norms for the measures completed shortly after diagnosis; (2) monthly distress screening also classified as mild, moderate, and high for mapping resources and recommendations; (3) these screening results and resource recommendations are provided to *the treating team and the family* and resources are available in the treatment center, online, and/or in their local community; (4) information on risk, distress, and mental health results are highlighted using color to convey severity of identified risk and mental health concerns (green = low risk, yellow = medium, red = high risk); finally (5) a key component of EPSI is the utilization of a health professional trained in psychosocial assessment and intervention in pediatric oncology called *the Psychosocial Navigator (PSN).* The term ‘navigator’ is used in other health care contexts where the role typically consists of assisting families to navigate the health system [34]. In the IG, the PSN serves as the contact person for families and the treating team, as the PSN provides feedback after the initial screening and monthly psychosocial distress screening follow-up for 12 months. Examples of resource recommendations by screening level are presented in Table 1. A copy of the Communication Summary Profile (CSP) with specific psychosocial service recommendations is presented in Fig. 3. An example of the monthly assessments and recommended resources that are communicated to the youth, caregiver, and treating team is presented in Fig. 4. EPSI intervention addresses several of the barriers previously identified by psychosocial health care providers (HCPs), including *how* to integrate screening results, limited knowledge of validated measures, and using the PSN we address the concern of the limited clinical resources that are available for conducting screening and ongoing monitoring of psychosocial needs [22].

**Table 1:** Examples of resource recommendations by risk level

Child/adolescent	Resources
*Universal/low*	Hospital volunteers
	Child life
	Look good feel better program
	Interlink nurses
	Hospital game room
	Borrowing distraction resources (e.g., games, audiobooks) from hospital resource center or local library
*Targeted/moderate*	Social work
	Web-based resources (https://youth.anxietycanada.com/, MindShift app)
	Mindfulness resources (in hospital or online; https://www.uclahealth.org/marc/mindful-meditations)
	Art, music, animal therapy
	Wellspring Cancer Support Center (https://wellspring.ca/)
	Camp Ooch in the city
	Upopolis (https://www.upopolis.com/login.html)
	Psychology consult
	(See universal/low resources)
*Clinical/high*	Psychology
	Psychiatry
	(See universal/low and targeted/clinical resources)
**Parent/caregiver:**	
Universal/low	Canadian Cancer Society web-based resources (http://www.cancer.ca/en/cancer-information/cancer-type/childhood-cancer-information/your-child-has-cancer/?region=on)
	POGO ( https://www.pogo.ca/programs-support/resource-guide/)
	SickKids resources guide for families facing childhood cancer ( https://www.sickkids.ca/PDFs/Haematology-Oncology/66601-oncology%20resource%20guide.pdf)
	Ontario Parents Advocating for Children with Cancer Parent Liaison
Targeted/moderate	Social work/resource navigators
	Mindfulness resources (in hospital or online; https://www.uclahealth.org/marc/mindful-meditations)
	Wheels of Hope
	Web-based resources (https://www.anxietycanada.com/adults/introduction ; MindShift app)
	Spiritual services (in hospital or community)
Clinical/high	Psychology
	Consult with general practitioner
	(See universal/low and targeted/clinical resources)

All participants complete the assessment measures at baseline and 12 months and participants in the IG group also complete the screening measure monthly (see below). At the 12-month follow-up, based on their preference, participants will either have survey packets mailed to them with pre-stamped envelopes to return completed surveys, or emailed to them via a secured link to REDCap. In the IG, personalized results from the psychosocial risk assessment and anxiety and depression assessment at baseline, and monthly distress screenings, are used to tailor resource recommendations for the youth and caregiver, which are shared with them and the youth’s treating team. The validated instruments are described below. Additionally, at 12 months, after completing the quantitative measures, the IG group participants complete a brief questionnaire of intervention acceptability and are invited to engage in a short semi-structured interview (approximately 15 min; see Table 3) where they are asked to describe their overall experience of the study. This interview is conducted either in person or over the telephone, depending on participant preference and availability. Interview data are audio-recorded and transcribed verbatim. The time line for the IG and CG is presented in Fig. 2.

### Assessments and outcome measures

The primary outcomes are feasibility and acceptability of the intervention. Secondary outcomes are measures of anxiety and depression symptoms and quality of life outcomes in the youth and caregiver, separately. Exploratory outcomes are measures of coping, social relationships, and physical functioning (pain, fatigue, and sleep quality). Demographic and clinical variables are also collected (see Table 2).

**Table 2:** Measures for both intervention and control group arms

Questionnaire	Measures	Caregiver self-report		Child/teen self-report		Parent-proxy report	
		Baseline	12-month F/U	Baseline	12-month F/U	Baseline	12-month F/U
^a^PAT	Psychosocial risk	X	X				
^a^PROMIS-anxiety	Anxiety	X	X	X	X		
^b^(PI-ED)	Anxiety and depression			X	X		
^a^PROMIS-Depression	Depression	X	X	X	X		
^b^PedsQL-generic	Quality of life (QOL) of child/teen			X	X	X	X
^b^PedsQL-cancer	QOL of child/teen (related to cancer)			X	X	X	X
^a^DT (intervention dyads also complete DT on a monthly basis)	Distress	X	X	X	X		
^b^PROMIS-fatigue	Fatigue	X	X	X	X		
^b^PROMIS-Emotional Support	Emotional support	X	X	X	X		
^b^PROMIS-Peer Relationships	Peer relationship support			X	X		
^b^Pittsburg Sleep Quality Index	Sleep quality	**X**	**X**	**X**	**X**		
^b^Response to Stress Questionnaire (RSQ-PC)	Coping with cancer diagnosis	X	X	X	X		
^b^Impact of Events Scale (IES-R)	Post-traumatic stress	X	X	X	X		
^b^Financial impact	Financial impact of diagnosis and treatment	X	X				
^c^Satisfaction questionnaire (intervention dyads only)	Satisfaction with intervention and study experience overall		X		X		

^a^Used for screening

^b^Used for exploratory purposes

^c^Used for acceptability

### Feasibility and acceptability

Outcomes and endpoints for feasibility are measured by criteria of intervention acceptability; recruitment and retention; intervention satisfaction; and feasibility of completing outcome measures of intervention effectiveness.

Acceptability will be assessed using (a) estimates of recruitment, retention, and withdrawal rate; (b) estimates of completed assessment screenings, data collection procedures, and specific outcome measures; (c) in the intervention group, youth’ and parents’ perceptions of the acceptability of EPSI intervention, including their acceptability of monthly screening and feedback by the PSN, as well as their views about the intervention gathered during the end of intervention interview. Finally, (d) examination of treatment effects (e.g., effect sizes, variance measures/standard deviation) on secondary mental health and quality of life outcomes and exploratory variables will inform the selection of outcome measures for the future, definitive RCT.

*Intervention acceptability* is measured by participation in screening, assessment, and intervention activities (defined as treatment accepters when the participant completes all required measures and questionnaires). Recruitment and *retention* will be measured by (i) accrual and dropout rates; and (ii) proportion of completed questionnaires (defined as 100% when all measures completed at baseline and 12 months post-baseline). *Intervention satisfaction* will be measured by (i) comparing recruitment and retention between both groups; (ii) brief questionnaire completed by all caregivers and youth at the completion of study; and (iii) by the semi-structured interview completed by youth and caregivers who participate in the IG (Table 3). Finally, potential benefits of EPSI will be estimated comparing the secondary outcomes (mental health and quality of life) and exploratory outcomes (coping, social relationships, and physical functioning) in the IG and CG.

**Table 3:** Semi-structured interview questions

1. **Thinking back to when you first agreed to participate in the study, what was it about the project that made you say “yes”?**	
2. **What was it like for you to participate in the study? (Tell us about your own experience?)**	
3. **What were your expectations about being in this study?**	
4. **How were your expectations met or not met? (Explain.)**	
5. **How did you find the experience with the Psychosocial Navigator?**	
6. **Do you have any additional comments or suggestions regarding the study?**	

### Data collection

#### All participants (IG and CG)

At baseline (2–4 weeks post-diagnosis), all measures are completed in person with the assistance of a research assistant and in a few cases, interpreter services (see Table 2). At follow-up (12 months post-baseline assessment), all measures are available to participant dyads to complete during clinic appointments days, at home with prepaid postage to return the surveys, orally via phone, or by email via a secured link to REDCap. Survey measures require approximately 30–45 min to complete (see Table 2).

#### Psychosocial risk

*Psychosocial risk* is measured using the Psychosocial Assessment Tool (PAT) revised for a Canadian sample [24]. The PAT consists of 7 psychosocial domains: family structure and resources; social support; child problems; sibling problems; caregiver stress reactions; family problems; and family beliefs. Scoring of the PAT items generates a 3-tiered psychosocial risk system derived from PPHM [16, 17]: *universal:* the family reports many supportive resources and relatively low psychosocial risk (typical of most families); *targeted:* the family reports some supportive resources but also some psychosocial risk factors, which may impact illness adjustment or treatment adherence (smaller group of families); and *clinical*: the family reports few supportive resources and multiple areas of difficulty (e.g., mental health in the family, financial problems, and social isolation) that may impede illness adjustment or treatment adherence (this is an even smaller group of families). Scores < 1 indicate universal risk, 1–1.9 represent targeted risk, and > 2 represent clinical risk. This measure is administered at baseline and 12 months only.

#### Mental health measures

*Anxiety symptoms* are measured using the Patient-Reported Outcomes Measurement Information System (PROMIS) [35]. The PROMIS-anxiety is an 8-item (pediatric) and 6-item (adult) measure with a 5-point Likert scale from 1 (never) to 5 (almost never). The items are added to provide a raw score which is then converted to a T-score. For the purpose of this study, a T score below 55 represent low symptoms, 55–64.9 represent moderate symptoms, and ≥ 65 represent high symptoms. This measure is administered at baseline and 12 months only.

*Depression symptoms* are measured using the Patient-Reported Outcomes Measurement Information System (PROMIS) [35]. The PROMIS-depression is a 6-item (pediatric) and 8-item (adult) measure with a 5-point Likert scale from 1 (never) to 5 (almost never). The items are added to provide a raw score which is then converted to a T-score. For the purpose of this study, a T score below 55 represent low symptoms, 55–64.9 represent moderate symptoms, and ≥ 65 represent high symptoms. This measure is administered at baseline and 12 months only.

#### Psychological distress

*Distress levels* are measured using the Distress Thermometer. The Distress Thermometer (DT) is a one-item, 11-point Likert scale represented on a visual graphic of a thermometer that ranges from 0 (no distress) to 10 (extreme distress). The adult/caregiver version has 15 accompanying checkboxes for areas of distress [36] while the pediatric version has 38 checkboxes for areas of distress [37]. The DT is reported to have sound psychometric properties [36, 37]. For the purpose of this study, scores below 4 represents low distress, 4–6 represents moderate distress, and ≥ 7 represents high distress. This measure is administered monthly.

#### Quality of life (QOL)

*QOL of child/teen* (based on self-report and caregiver report) are measured using the PedsQL 4.0–generic and PedsQL 3.0–cancer. PedsQL 4.0 is a 23-item scale that provides a total and four subscale scores (physical, social, emotional, and school functioning) [38]. It has adequate test-retest reliability and validity. PedsQL 3.0 is a 24-item cancer-specific scale with a total and eight subscales including: pain, nausea, procedure, and treatment anxiety [39]. Raw scores in both scales are linearly transformed to a 0 to 100 scale (higher scores reflect better QOL). The PedsQL-3.0 has adequate test-retest reliability and validity [39]. Scores below 69 are considered poor QOL [40].

*Caregiver QOL* is assessed using the Caregiver Quality of Life Cancer Scale*,* a 35-item questionnaire that assesses QOL globally and across four domains: burden, positive adaptation, disruption, and financial; lower scores indicate better quality of life [41]. The validity and reliability of this measure is adequate.

#### Social relations and coping

*Socio-emotional support* will be measured using the PROMIS. The PROMIS-emotional support is a 7-item (pediatric) and 6-item (adult) measure with a 5-point Likert scale. T scores below 30 represents poor peer relationships, 30–40 represent fair peer relationships, 40–60 represent good peer relationships, and ≥ 60 represents excellent peer relationships.

*Peer relationship support* is measured using the PROMIS. The PROMIS-peer relationship is an 8-item (pediatric) measure with a 5-point Likert scale. T scores below 30 represent poor peer relationships, 30–40 represents fair peer relationships, 40–60 represents good peer relationships, and ≥ 60 represents excellent peer relationships.

*Coping* with cancer diagnosis is measured using the Responses to Stress Questionnaire-Cancer version (RSQ-PC). The RSQ-PC is a measure of coping and involuntary stress responses. The factor structure of the RSQ has been supported in confirmatory factor analysis studies with children and adolescents from a wide range of ethnic and cultural backgrounds coping with a variety of stressors [42]. The coping scales include primary control coping (i.e., problem solving, emotional modulation, emotional expression), secondary control coping (i.e., acceptance, cognitive restructuring, positive thinking, distraction), and disengagement coping (i.e., avoidance, denial, wishful thinking). Using the standard method for scoring the RSQ, and to control for response bias and individual differences in base rates of item endorsement, proportion scores are calculated by dividing the total score for each factor by the total score for the RSQ [43].

#### Physical measures

*Sleep quality* is assessed using the Pittsburgh Sleep Quality Index (PSQI). The PSQI is a self-rated questionnaire which assesses sleep quality and disturbances over a 1-month time interval. Nineteen individual items generate seven “component” scores including subjective sleep quality and sleep duration. The sum of scores for these seven components yields one global score; scores greater than 5 indicates poor sleep quality. The PSQI is reported to have acceptable psychometric properties [44].

*Fatigue symptomatology* is measured using the PROMIS. The PROMIS-fatigue is a 10-item (pediatric) and 6-item (adult) measure with a 5-point Likert scale. The items are added to provide a raw score which is then converted to a T-score. For the purpose of this study, a T score below 55 represents low symptoms, 55–64.9 represents moderate symptoms, and ≥ 65 represents high symptoms.

*Pain-related quality of life* is measured via the pain subscale of the PedsQL (see above).

### Data analysis

#### Quantitative data analysis

Preliminary analyses will include descriptive statistics for key study variables of feasibility. First, frequencies and percentage rates will be collected for measures of feasibility such as recruitment, retention, and withdrawal. Consistent with previous research [33, 45, 46], the intervention will be feasible if at least 50% of newly diagnosed families are recruited to the study, 80% of the targeted sample size (n = 40) is recruited, at least 70% of the sample is retained, and fewer than 30% of participants withdraw from the study. The intervention will be acceptable if at least 75% of the IG rate the intervention as ‘pretty much’ or ‘very much’ acceptable.

Preliminary t tests on the secondary and exploratory outcome measures will be conducted to determine which outcome measures should be further examined in the larger multi-site trial.

#### Qualitative data analysis

After the semi-structured interviews are transcribed verbatim, they will be analyzed using inductive content analysis [47]. Analysis will consist of an iterative, open coding process where two primary coders will immerse themselves in approximately five transcripts. Recurring themes and relational patterns common across transcripts will be explored with the aid of charts and notes. The coders will meet regularly to compare notes and develop codes, keeping a paper trail throughout analysis [48]. Discrepancies will be resolved by consensus. Following open coding, categories, themes and subthemes will be grouped together to form a codebook with all codes defined. The primary coders will then code all transcripts using the codebook and MAXQDA software to facilitate the data analysis. Ten percent of interviews will be double coded by a third researcher to ensure acceptable (> 85%) inter-rater reliability.

#### Data management

Data is stored in line with HSC ethical procedures and requirements for storage and security of data. Participant questionnaires are identified with a numeric code and kept separate from all identifiable personal information such as names, addresses, and/or consent forms. Data quality will be checked using double data scoring and entry. Standard data management protocols are being followed. Only the study investigators and research assistants will have access to the study data. Ethics auditing procedures are determined by the institutional research ethics board.

#### Safety monitoring and reporting

Should a participant report risk to themselves or others, the research team will take all necessary steps to ensure the safety of the participant and others. All risks or safety issues will be reported to the Principal Investigator who will take any necessary further steps and provide ongoing clinical supervision to the research team as required. Additionally, data safety and monitoring board guidelines will be followed.

Debriefing sessions and clinical supervision with the Psychosocial Navigator (the research team member conducting the intervention) will take place regularly.

Any serious adverse events will be recorded and reported to the institutional ethics committee.

#### Ethical approval

The study received ethical approval from the Hospital for Sick Children Research Ethics Board on 2 November 2017 (REB # 1000056445). The trial will be conducted in accordance with the Ethics Approval at all times. The participants will be identified by a study-specific participant number in all databases. Any amendment to the protocol will be submitted to the research ethics board for approval.

#### Dissemination

The following knowledge translation activities will be conducted with the intention of advancing the field of screening for psychosocial risk and mental health in pediatric oncology: delivery of scientific rounds at the participating centers to disseminate relevant results for stakeholders in pediatric oncology as well as other pediatric chronic conditions; training workshops on the implementation of the intervention provincially and nationally; and participation in community presentations for parent organizations (e.g., Ontario Parents Advocating for Children with Cancer) and scientific presentations provincially (Pediatric Oncology Group of Ontario, POGO Symposium), nationally (Canadian Psycho-Oncology meetings, CAPO), and internationally (International Pediatric Oncology Association, SIOP).

## Discussion

A large barrier to implementation of screening programs is a lack of knowledge on *how* to follow-up from screening. This proposal describes a new intervention designed to bridge the gap between psychosocial screening and better health outcomes. By tailoring recommendations and resources to the assessment results initially and providing ongoing monthly feedback via the psychosocial navigator, we hope to capture potential changes in the psychosocial needs of the youth and the primary caregiver and improve their mental health outcomes. This proposal addresses the process of assessing feasibility and acceptability of the intervention and pilot testing of its possible benefits for the child and the primary caregiver using a randomized control trial. It is designed to meet recommended standards of care [14] and builds on extensive previous work in adult oncology [49] and preliminary findings in pediatric oncology [21, 26, 27].

Positive findings of feasibility and acceptability of the intervention will serve as the foundation for an RCT fully powered to test intervention effects. Broadly, this intervention may lead to earlier identification of families at-risk for poor quality of life and poor mental health and more efficient and precise triaging of resources based on measured risk, mental health, and distress in the youth with cancer and their family. This intervention has the potential to model a pathway forward on how to implement psychosocial screening in pediatric oncology. If successful, this intervention could then be expanded across other pediatric chronic conditions.

### Trial status

Recruitment began in November 2017. Thirty-eight dyads have been enrolled. We anticipate data collection will be completed by October 2020.

## Data Availability

Not applicable.

## Abbreviations

CSP: Communication Summary Profile
CG: Control group
DT: Distress thermometer
EPSI: Enhanced Psychosocial Screening Intervention
HCP: Healthcare provider
HSC: Hospital for Sick Children
IG: Intervention group
PAT: Psychosocial Assessment Tool
PPPHM: The Pediatric Preventive Psychosocial Health Conceptual Model
PROMIS: Patient-Reported Outcomes Measurement Information System
PSN: Psychosocial navigator
PSQI: Pittsburgh Sleep Quality Index
QOL: Quality of life
RCT: Randomized Controlled Trial
RSQ-PC: Response to Stress Questionnaire-Pediatric Cancer
